# Supplementary material for: Neuroimaging markers of Alice in Wonderland syndrome in patients with migraine with aura
Source: Front Neurol. 2023 Aug 24;14:1210811. doi: 10.3389/fneur.2023.1210811 (PMC10520557; doi:10.3389/fneur.2023.1210811)
Supplement: Supplementary file 1 [file Table_1.docx]

| N | Sex | Age | Age at onset | Headache frequency (days/month) | Duration of AIWS episodes | Description of AIWS symptoms | AIWS type |
| --- | --- | --- | --- | --- | --- | --- | --- |
| 1 | F | 45-49 | 25-29 | 6 | Up to 4 hours | Macrosomatognosia of face and upper limbs, mosaic vision, derealization, depersonalization, slowing in the perception of time | Somatosensory + Visual  (type C) |
| 2 | F | 55-59 | 20-24 | 20 | 20-30 minutes | Micropsia, derealization, depersonalization, slowing in the perception of time | Visual  (type B) |
| 3 | F | 15-19 | 15-19 | 25 | 10 minutes | Macropsia, telopsia, derealization, depersonalization, slowing in the perception of time | Visual  (type B) |
| 4 | F | 45-49 | 5-9 | 2 | 15 minutes | Microsomatognosia, telopsia, pelopsia, derealization, depersonalization | Somatosensory + Visual  (type C) |
| 5 | M | 25-29 | 10-14 | 8 | Up to 1 hour | Telopsia, pelopsia, mosaic vision, slowing in the perception of time | Visual  (type B) |
| 6 | F | 45-49 | 15-19 | 5 | Not reported | Macrosomatognosia of hands, micropsia, macropsia, telopsia, pelopsia, derealization, depersonalization, slowing in the perception of time | Somatosensory + Visual  (type C) |
| 7 | M | 35-39 | 20-24 | 10 | Not reported | Micropsia | Visual  (type B) |
| 8 | F | 55-59 | 15-19 | 2 | Up to 1 hour | Micropsia, macropsia, derealization | Visual  (type B) |
| 9 | F | 40-44 | 10-14 | 10 | 30 minutes | Macrosomatognosia left hemiface, macropsia, pelopsia | Somatosensory + Visual  (type C) |
| 10 | F | 45-49 | 45-49 | 3 | 30 minutes | Macrosomatognosia of hands and upper limbs, aschematia, macropsia, telopsia, mosaic vision, derealization, depersonalization, slowing in the perception of time | Somatosensory + Visual  (type C) |
| 11 | F | 25-29 | 10-14 | 3 | 1 hour | Aschematia, mosaic vision, derealization, depersonalization, slowing in the perception of time | Visual  (type B) |
| 12 | M | 40-49 | 25-29 | 4 | Not reported | Pelopsia, mosaic vision, loss of stereotactic vision, slowing in the perception of time | Visual  (type B) |
