# Supplementary material for: Neuroimaging markers of Alice in Wonderland syndrome in patients with migraine with aura
Source: Front Neurol. 2023 Aug 24;14:1210811. doi: 10.3389/fneur.2023.1210811 (PMC10520557; doi:10.3389/fneur.2023.1210811)
Supplement: Supplementary file 2 [file Table_2.docx]

| N | Sex | Age | Age of onset | Headache frequency (days/month) | Aura characteristics |
| --- | --- | --- | --- | --- | --- |
| 1 | F | 20-24 | 15-19 | 2 | visual + somatosensory |
| 2 | M | 40-44 | 20-24 | 10 | visual |
| 3 | M | 20-24 | 5-9 | 3 | visual |
| 4 | M | 40-44 | 5-9 | 10 | visual |
| 5 | M | 50-54 | 10-14 | 5 | visual |
| 6 | F | 35-44 | 10-14 | 3 | visual |
| 7 | F | 60-64 | 15-19 | 1 | visual + somatosensory |
| 8 | M | 25-29 | 25-29 | 6 | visual |
| 9 | F | 20-24 | 10-14 | 10 | visual |
| 10 | F | 20-24 | 15-19 | 1 | visual + somatosensory |
| 11 | M | 35-39 | 10-14 | 3 | visual |
| 12 | F | 20-24 | 5-9 | 1 | visual |
