# Supplementary material for: Neuroimaging markers of Alice in Wonderland syndrome in patients with migraine with aura
Source: Front Neurol. 2023 Aug 24;14:1210811. doi: 10.3389/fneur.2023.1210811 (PMC10520557; doi:10.3389/fneur.2023.1210811)
Supplement: Supplementary file 3 [file Table_3.docx]

|  | MA | | HC | |
| --- | --- | --- | --- | --- |
|  | RRR | p | RRR | p |
| Thal - Posterior precuneus | 0.336 | 0.104 | 0.008 | 0.02 * |
| Thal - Calcarine cortex | 0.558 | 0.226 | 0.003 | 0.047 * |
| V3 - STS | 0.476 | 0.012 * | 0.576 | 0.286 |
